# Homœopathic Literature

**Published:** 1843-06-10

**Authors:** 


### THE MEDICAL EXAMINER.

PHILADELPHIA, JUNE 10, 1843.

### HOMEOPATHIC LITERATURE.

"The Homeopathic Medical Library," is proposed to be "put to press as soon as a sufficient number of subscribers is obtained to warrant it." This fact we learn from a copy of the Prospectus and a specimen of the work now before us.

This undertaking is required on account of the "acknowledged imperfections of the text books now in use."

We notice this undertaking in hopes of showing the profession the kind of reasoning, and also some of the remarkable fallacies of the so-called Science of Homeopathy.

We are first told that all "diseases consist of a number of symptoms of greater or less extent, and constitute the affections presented to us in the *high sounding* and *classical,* but *deceptive language* of nosology; which conveys simple facts with a *high flourish of inflated learning,* to prevent the cognition of the uninitiated reader, in place of the plain unsophisticated language of true philosophy, more intelligible to the mind, and far more useful in practice." When stated in plain language, we presume the writer means to say, that medical authors of the Allopathic School purposely write so as not to be understood by the uninitiated reader. "La raison de vos des raisons affablie tant ma raison que c'est ne pas sans raison que je me plain de votre beauté."

We are next told that medicines in the healthy body produce respectively, "the very groups of symptoms that are constantly observed in disease," and that any "physician *whose mind has not been perverted by education*" ("learning *is* a dangerous thing,") may be certain that "medicinal diseases or pathogenesis" and pathology "are intended for each other in the character of evil and remedy." "This fact, however," "would really seem to have escaped the notice of some of our most reputed authors in Homeopathic literature; for it appears to the Editors, that the Materia Medica of JAHR" "is a *gross perversion*" "of the text of HAHNEMANN."

Consequently, as the Materia Medica of Jahr is a gross perversion of Hahnemann, some of the best authors in Homeopathy have failed to discover that pathogenesis and pathology are intended for each other in the character of evil and remedy. This is certainly a remarkable deduction to be drawn from the dishonesty (alleged) of Jahr!

According to the Editors of the "Homeopathic Medical Library," the work of Jahr is not only a gross perversion of fact, but the English version "is extremely imperfect," "and also a perversion of the original," consequently say they, "it will be easy to perceive that our literature *for practical use,* is in our own language, alarmingly on the decline," and "it is high time to have a careful translation from the fountain-head, to guard the practitioner against the errors of authors, who may be more influenced in their labours by sordid considerations than by a love of Homeopathy."

Connected with the Homeopathic Materia Medica, the Editors give the following curious information:

"It may be well also to advert to the fact, that some cunning wags, surcharged with pathological lore have indulged their hostility to the science, in the fabrication of the pathogenesis of some new and probably useless remedies, and have unaccountably succeeded in imposing on the credulity of those who have been in a measure regarded as the sentinels of our system, and thereby obtained their publication to the world, connected with authentic literature. These, however, are counterfeits; easily detected by those acquainted with the general character of a genuine pathogenesis, and furnish us a wholesome precept to regard with suspicion what does not emanate from an authentic source. Those in possession of JAHR's former edition, may notice *alkekengi* and several other remedies as examples of this fatal and disgraceful imposition."

These remarks upon the work of Jahr are important, because it has been very extensively relied upon by Homeopathic Doctors, and if the author could be so far hoaxed, as to describe as a medicine what existed only in the brains of "cunning wags," his powers of reasoning and observation are questionable, as well as his honesty, which is very obliquely spoken of by our Editors.

Although Homeopathic literature in the English language is made up of perversions and imperfections, our Editors admit there is something better in the old school. They declare that,

"Pathology cannot be better studied than in the ample and learned works of the old school; where the intelligent practitioner despises the learned lumber of his profession as much as we do, and when at the bed-side, is plain and intelligible. The only thing the Homeopathic student has to avoid in their works, is that part of the story, which is generally very short, and, according to the orthodox school, is easily told, which informs us what is to accomplish the great work of curing the disease. The rest is all in a state of great refinement, and belongs as much to the education of the intelligent practitioner of specific medicine, as of the pertinacious stickler for constitutional principles; which, in their application, are no less at variance with the integrity of the constitution of the human body, than are some principles prescribed by high operative political physicians with that of the Constitution of our country; both are too much on the *taxing* principle."

For the surprising effects of medicine and its economical use, we must look to Homeopathic writers, of whose credibility, it is presumed, there can be no doubt; and to sustain this presumption, we quote from the specimen of the "Homeopathic Medical Library" a part of the account given of Aconite by the great Hahnemann:

"Every time that Aconite is chosen as a Homœopathic remedy, it is especially necessary to regard the moral symptoms, and be careful that they resemble those which belong to it.

Aconite is indispensable for females who suffer from fear or contrarieties during the catamenia; for without this precious calmer, it happens indeed too often, that they suffer even a sudden arrest from such moral shocks. In such cases it will be sufficient to direct a single inspiration, and for an instant, from a bottle containing a globule of the size of a mustard seed, which has been impregnated with the thirtieth dilution; and this will preserve its virtue some years without any loss, provided the bottle is always well closed."

If this statement can be incontestably proved, there is no doubt but Homeopathy would entirely supercede Allopathy; medical schools would be closed, and all we should require to be able to treat disease would be to read the Homeopathic Medical Library.

How long will people delight in being deceived and cajoled by shameless charlatanry. There is nothing in astrology or palmistry more unworthy of sober consideration than this same subject of Homeopathy; and the only inducement to notice such trash is to discharge a duty towards the credulous and "uninitiated reader." It is among the duties of public Journalists to correct errors, and point out falsehood, and denounce it to the best of their power. And we cannot better close these remarks than by declaring our opinion that practitioners have made money and obtained notoriety by Homeopathy, not because there is any truth in Homeopathy, but because of the gullibility of the people. If its being in fashion proved its truth, we have a right to claim the same truth for Perkin's Metallic Tractors, for the efficacy of amulets and charms, and even for Astrology.

